# Androgens in endometrial carcinoma: the killer or helper?

**DOI:** 10.1007/s40618-022-01916-1

**Published:** 2022-12-30

**Authors:** X. Wu, K. Zhang, X. Zhong, X. Huo, J. Zhang, W. Tian, X. Yang, Y. Zhang, Y. Wang

**Affiliations:** 1 Department of Gynecology and Obstetrics, Tianjin Medical University General Hospital, Tianjin, China; 2 Department of Gynecology and Obstetrics, Affiliated Hospital of Nantong University, Nantong City, China

**Keywords:** Androgens, Metabolism, Androgen receptor, Endometrial carcinoma, Risk–benefit

## Abstract

**Purpose:**

The aim of this review is to discuss the role of androgens in the progression of endometrial carcinoma (EC) with particular focus on the different kinds of androgenic hormones, androgen receptor (AR) and intracrine androgen metabolism.

**Methods:**

A comprehensive literature search within PubMed was performed. Selected publications related to androgens and EC were reviewed.

**Results:**

There are different kinds of androgenic hormones, and different kinds of androgens may have different effects. Elevated androgens (especially testosterone) have been associated with an increased EC risk in postmenopausal women. 5α-reductases (5α-Reds) and 17β-hydroxysteroid dehydrogenase type 2 (17βHSD2) pathway may inhibit the progression of EC mediated by dihydrotestosterone (DHT), but aromatases stimulate further progression of EC. The most of studies accessing the prognostic value of AR have found that AR expression may be a favorable prognostic indicator.

**Conclusion:**

Androgens may have both oncogenic and tumor suppressive roles. Androgen-specific biases in metabolism and the expression of AR may contribute to the different prognosis of patients with EC.

## Introduction

Endometrial carcinoma (EC) is the most commonly diagnosed gynecological cancer in women and ranked second in gynecological cancer-related deaths [1]. Risks for EC are associated with an excess-estrogen environment. And the role of unopposed estrogen in EC has been well described. To our best knowledge, one of the estrogen productions is aromatization of androstenedione to estrone within the ovaries and extragonadal tissues. Of note, the main source of estrogen production in women after menopause is from peripheral aromatization in adipose tissues [2]. All of these suggest the potential of androgens in the carcinogenesis of EC. In this review, we particularly focus on observational and mechanistic studies assessing the effects of androgens in women with EC. And the mechanistic studies mainly include the metabolism and androgen receptor that are most studied. We summarize some of the recent insights into androgen-regulated pathways that may pave the ways for untreatable cancers such as EC, at present.

## Androgens and the risk of EC

Endometrial growth is mediated by circulating sex hormones including androgens. There are several kinds of hormones that can exert androgenic effects, including dehydroepiandrosterone-sulfate (DHEAS), DHEA, androstenedione, testosterone and dihydrotestosterone (DHT) in the order of high to low level. Among them, DHT exerts the most potency of androgen, while androstenedione and DHEA exhibit only 10 and 5% of the potency of testosterone [3].

Some studies have examined the potential role of circulating androgens as risk factors for EC. In general, elevated serum testosterone level has been associated with an increased EC risk in postmenopausal women (Table 1). However, the association between increased testosterone levels and EC risk has not been observed among premenopausal women. Interesting, the association of androgens with EC risk is weaker when the levels of serum estrogens are taken into account [4, 5], suggesting that the conversion of testosterone into estrogen may contribute to the association between androgen and EC risk. An excess-estrogen environment is specially associated with type I EC, but few significant difference was revealed when type I cancer patients compared with type II cancer patients [6].

**Table 1:** Association between serum testosterone levels and EC risk in prospective studies

Reference	Cases	Controls	Testosterone levels in cases as a percent of control (*P* value)	OR (*P* value)	Androstenedione levels in cases as a percent of control (*P* value)	OR (*P* value)	DHEA levels in cases as a percent of control (*P* value)	OR (*P* value)	DHT levels in cases as a percent of control (*P* value)	OR (*P* value)
Postmenopausal women										
AM et al. 2021 [46]	549	159,153	> 100 (NA)	2.41^a^ (< 0.001)	–	–	–	–	–	–
Fei Teng et al. 2020 [48]	353	339	114 (< 0.001)	1.791^b^ (0.001)	138 (< 0.001)	0.959^a^ (0.826)	–	–	–	–
Kara A. Michels et al. 2019 [5]	313	354	117 (0.001)	1.91^c^ (0.01)	108 (0.02)	2.36^b^ (0.01)	110 (0.10)	1.85^b^ (0.07)	100 (0.34)	1.04^b^ (0.74)
Audet-Walsh E et al. 2011 [6]	126	110	164^d^ (< 0.05)	11.83^e^ (< 0.05)	155^c^ (< 0.05)	10.75^d^ (< 0.05)	157^c^ (< 0.05)	9.52^d^ (< 0.05)	125^c^ (< 0.05)	7.36^d^ (< 0.05)
Allen NE et al. 2008 [56^]^	192	374	110 (< 0.05)	1.44 (0.14)	99 (> 0.05)	0.86 (0.52)	–	–	–	–
Lukanova A, et al. 2004 [7]	124	236	122 (0.04)	2.06 (0.02)	133 (0.01)	2.15 (0.04)	–	–	–	–
Premenopausal women										
Clendenen TV et al. 2016 [57]	161	303	109 (0.04)	1.59 (0.08)	102 (0.54)	1.07 (0.80)	–	–	–	–
Allen NE et al. 2008 [56]	55	107	102 (> 0.05)	–	103 (> 0.05)	–	–	–	–	–

*NA* Not applicable

^a^Addjusted for age and other circulating hormone

^b^Adjusted for insulin, estrone, BMI, WHR, and family history of cancer

^c^Adjusted for gravidity, smoking status, body mass index, duration of oral contraceptive use, and age at menarche. If the OR was adjusted for unconjugated estradiol, the significance of four androgen hormones diminished

^d^Type I EC vs healthy control

^e^Adjusted for age and BMI

The increased EC risks with androstenedione (A4) were also found in most studies after menopause (Table 1). After adjustment for estrogen, the positive association was attenuated but the directions of the effect were unchanged [5, 7]. These results indicate that androgens affect endometrium carcinogenesis through not only conversion into estrogen but also androgenic pathways.

**Fig. 1:**
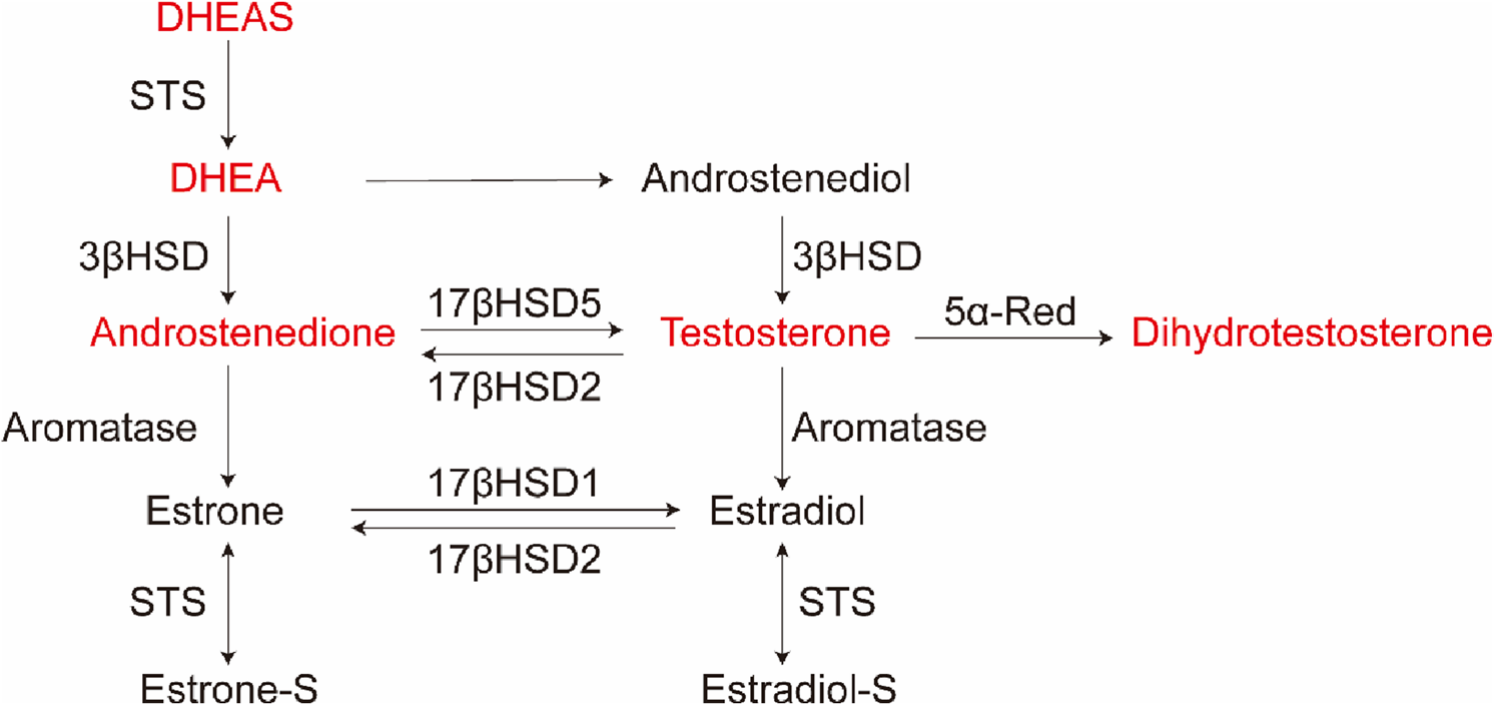
Selected aspects of androgen metabolism in endometrial cancer. The five androgen hormones are in red. *DHEAS* dehydroepiandrosterone-sulfate, *DHEA* dehydroepiandrosterone, *STS* steroid sulfatase, *A4* Androstenedione, *T* Testosterone, *DHT* Dihydrotestosterone, *17βHSD2* 17β-hydroxysteroid dehydrogenase type 2, *17βHSD5* 17β-hydroxysteroid dehydrogenase type 5, *5α-Red* 5α-reductases

To date, very few studies focused on the dihydrotestosterone (DHT), more potent than testosterone, in the role of EC risk. DHT is metabolized by 5α-reductase pathway and the reaction is not reversible (Fig. 1). Existing studies have not definitively indicated an increased EC risk associated with DHT levels. However, it is worth to note that a reduced risk for EC was associated with increasing DHT levels especially after adjustment for estrogen [5].

The link between endometrial safety and androgen treatment has been investigated in postmenopausal women treated with androgens for diminished sexual wellbeing. Recent data form a meta-analysis showed that androgen treatment did not increase endometrial thickness in postmenopausal women [8]. However, the included studies in this meta-analysis had a relatively short follow-up period (˂ 2 years), which may not be sufficient to observe serious adverse endometrial effects. The effect of exogenous testosterone treatments on endometrium tissues has also been studied in transsexuals. No morphological changes associated with EC risk have been observed in hysterectomy tissues [9]. Given that gender reassignment surgery-hysterectomy usually occurs relatively soon (˂ 3 years), the morphological changes could not be observed, limiting the utility of the data. In female-to-male (F-to-M) breast tissues, it has been observed that expression of breast cancer-related genes overlaps with these in F-to-M breast tissues [10]. It is tempting to investigate whether or not changes in cancer-related gene expression have existed.

## Androgens metabolism and EC

Once inside the cells, androgens and their precursors can be further metabolized and converted into bioavailable androgen in specific tissues containing the appropriate enzymes [2, 11]. In target tissues, the most of active androgens are produced locally from circulating adrenal and/or ovarian precursors [12, 13]. Differences in intracrine pathway are now accepted as a key way in which target tissues such as endometrium can respond to physiological demands. This may have the potential to confound the interpretation of studies investigating the association between androgen and EC. Therefore, it is important to elucidate the metabolic pathways that modulate intracellular levels of androgen in the endometrium. The expression of metabolic enzymes that have been implicated in EC are described in Table 2.

**Table 2:** Expression of related enzymes in cancerous tissues and EC cell lines

	Cell types	EC tissue (method)	References
STS	–	Yes (RT-PCR, WB, IHC)	[14, 15]
3βHSD	–	Yes (RT-PCR)	[18]
17βHSD1	–	Yes (RT-PCR, IHC)	[19, 20]
17βHSD2	HEC-1B, HEC-1A	Yes (RT-PCR, IHC)	[18, 24, 58]
17βHSD5	HEC-1A, Ishikawa	Yes (RT-PCR, IHC)	[18, 25, 59, 60]
5α-Red	HEC-1A, Ishikawa	Yes (RT-PCR, WB, IHC)	[26, 29, 61]
Aromatase	–	Yes (RT-PCR, WB)	[15, 29]

### Steroid sulfatase

One of the primary roles of steroid sulfatase (STS) is to hydrolyze DHEAS to DHEA. Also, STS acts on substrates like estradiol-S and estrone-S. STS mRNA and protein levels were detected both in cancer tissues and normal endometrium, but no significant difference was found [14, 15]. A larger cohort consisting of 175 tumor samples suggests that high STS levels are more associated with endometrioid histology which predicts a better prognosis but also with high grade (G2 and G3) which means poor prognosis [16]. Interesting, the clinical outcomes were also influenced by the STS/estrogen sulfotransferase expression ratio. Increasing this ratio predicted a poor outcome [17]. In EC tissues, the presence of STS may be more involved in estrogen supplement [14, 15]. Whether or not STS expression alters androgen levels in EC is still not known.

### 3β-Hydroxysteroid dehydrogenase

3β-Hydroxysteroid dehydrogenase (3βHSD) enzymes catalyze the conversion of DHEA to A4 and androstenediol to testosterone. Little is known about it in the role of EC, with only one study suggesting no significant changes in EC compared with normal tissues [18]. Correlations between 3βHSD expression and clinic-pathological markers have not been observed. And the correlation between 3βHSD and androgens is still unknown.

### 17β-Hydroxysteroid dehydrogenase type 1

The most potent estrogen estradiol (E_2_) is produced via 17β-hydroxysteroid dehydrogenase type 1 (17βHSD1). To date, the role of 17βHSD1 in EC has been investigated more in relation to the production of estrogen rather than androgen. An increase in the levels of 17βHSD1 has been shown to be associated with increased levels of E_2_ in EC [19, 20]. Estrogen is a well-established risk factor for EC, since it could drive EC progression [21, 22]. A decrease in levels of 17βHSD1 has been shown to be associated with better prognosis [16]. However, similar to STS, the function of 17βHSD1 may more contribute to the regulation of estrogen action in this disease. Whether or not expression of 17βHSD1 in EC influences androgen actions is needed to study further.

### 17β-Hydroxysteroid dehydrogenase type 2

17β-Hydroxysteroid dehydrogenase type 2 (17βHSD2) is responsible for generating A4 from testosterone and the reversible conversion between estrone and estradiol. Studies assessing the clinical significance of 17βHSD2 in EC have suggested conflicting findings. A relatively well-designed cohort study identified that 17βHSD2 was significantly higher in tumors compared with normal tissues, especially in endometrioid type II cancers [23]. Furthermore, survival analyses confirmed that patients with high level of 17βHSD2 had a better prognosis than the remaining women [16, 24]. In EC, interactions between androgens and 17βHSD2 have been suggested. A study on HEC-1B cells treated with DHT, which were inhibited growth, indicated that anti-proliferative effects of DHT were mediated through an increase in 17βHSD2 levels [24]. This is consistent with the finding from an observational study that increasing DHT level reduced risk for EC occurrence [5]. These lines of evidence suggest that the tumor-suppressive role of androgens may be partly attributed to the expression of 17βHSD2. However, this hypothesis is yet to test in a clinical setting.

### 17β-Hydroxysteroid dehydrogenase type 5

In contrast to the role of 17βHSD2, 17β-hydroxysteroid dehydrogenase type 5 (17βHSD5) is involved in the conversion of A4 into T.

To date, 17βHSD5 expression in cancer tissues as opposed to normal endometrium is still uncertain. The largest published study identified a decreased 17βHSD5 level measured by immunohistochemistry (IHC) in 123 EC specimens compared with adjacent normal endometrium. Further survival analysis revealed that increased expression of 17βHSD5 in endometrioid endometrial cancer was associated with better cumulative and disease-specific survival [25]. 17βHSD5 can convert A4 to T. Therefore, the lower 17βHSD5 expression in EC may contribute to T loss in the diseased endometrium. One study examined the intratumoral T concentration of EC, but did not find significant difference between the intratumoral concentration of T in EC and the normal endometrium [26]. The small numbers of patients included make definitive conclusions difficult. Therefore, there is clearly a need for well-designed, larger numbers included studies to uncover the role of 17βHSD5-mediated androgens conversion.

### 5α-Reductases

5α-Reductases (5α-Reds), which have three different isoforms-5α-Red1, 5α-Red2, and 5α-Red3, catalyze the conversion of testosterone to DHT. In EC, 5α-Red1 is the most widely investigated among them. 5α-Red1 was considered the main isoform in EC tissues and expressed more frequently than 5α-Red2. And the levels of 5α-Red1 expression were upregulated compared with matched tissues [27–29]. However, the increased DHT concentration within cancerous tissues was not observed. It is noteworthy that 5α-Red1 was an independent prognostic factor in EC, where its expression was significantly associated with lower tumor grade and progression-free survival (PFS) in positive group was longer than that in negative group. Additionally, 5α-Red1 rather than 5α-Red2 was positively associated with intertumoral DHT, suggesting its higher affinity for testosterone than 5α-Red2 [26]. Combined with the results from clinical data [5], it is tempting to suggest us that the conversion of testosterone to DHT via 5α-Reds may weaken the oncogenic role of testosterone. Further studies are needed to identify it.

5α-Reds, especially 5α-Red1, are also involved in progesterone metabolism. As we know, progesterone can counteract the effect of estrogens within the endometrium. The suppressive effect of 5α-Red1 may be involved in androgens and/or progesterone actions in endometrium tumorigenesis, indicating the need for a better understanding of it to better define the role of 5α-Red1 in EC.

The importance of 5α-Red2 and 5α-Red3 in EC is known little. Deceased levels of 5α-Red2 and increased 5α-Red3 expression were detected in EC [27, 30], although significant effects of 5α-Red2 and 5α-Red3 on prognosis have not been reported. Further studies are needed to be done to understand the specific role of 5α-Red2 and 5α-Red3 in EC.

### Aromatase

The aromatases encoded by CYP19A1 gene catalyze the last steps of estrogen biosynthesis, which stimulates the cell proliferation in EC. This forms the basis for aromatase inhibition as a therapeutic strategy for EC. When aromatase inhibitors (AIs) reduce the peripheral conversion of androgens to estrogen, the levels of androgens increase in peripheral circulation and/or EC tissues. It has been reported that DHT could inhibit the proliferation of EC cells induced by estrogen [31]. Therefore, it is tempting to speculate that the findings of increased EC risk associated with androgen levels are largely the result of conversion to estrogen by aromatase in endometrium. As shown in Table 1, one study has evaluated the connection influenced by estrogen and found that the effect of elevated testosterone on EC risk is ameliorated when the conversion of testosterone to estradiol is taken into account [4]. Further interactions between androgen signaling and aromatase have not been suggested at the molecular level. However, there is no association between mRNA levels of aromatase and serum testosterone [32]. Given the sample size of this study, there is clearly a need for well-designed, larger scale studies to understand the mechanism in it.

## The role of androgen receptor in normal endometrium

Evidence that the endometrium is an androgen-target tissue is supported by detection of AR in the tissue [33]. Normal adult endometrium including epithelial and stromal cells detected the expression of AR. And AR expression was more abundant in the stromal cells during the proliferative phase of the menstrual cycle. As for epithelial cells, AR was nearly undetectable in the proliferative phase, while weak in the secretory phase [28, 34]. Interestingly, AR may play both a growth stimulatory and inhibitory role. The primary role of epithelial AR appears to induce epithelial cell apoptosis. Stromal AR may function as stimulator of endometrial cells proliferation [35].

AR knockout (ARKO) mice are important for the study of AR in EC. In these animals, normal but smaller uteri and reduced uterine growth at oestrous or in response to exogenous gonadotrophins are found. Besides, AR appears to involve in the development of pups and placental. The number of pups per litter is significantly decreased before ovarian failure exists in ARKO mice. Placentomegaly and abnormal placental development are more frequent during pregnancy in these animals [36].

## The role of AR in EC

Traditionally, EC has been categorized as type I or type II based on histology. In type II EC, high-level AR expression was restricted to serous cancer in concert with ER and PR expression. Although this study did not provided data on the association between AR and EC outcomes, the patients with AR-positive malignancies were older than those with AR-negative tumors [37]. AR staining was not detected within uterine carcinosarcoma, endometrial stromal sarcoma, or clear cell cancers [38, 39]. Of rare histologic types like undifferentiated/dedifferentiated carcinomas, the presence of AR has been reported. Loss of AR indicates the possibility of more advanced disease [40].

Type I endometrioid adenocarcinomas comprise most cases. Most of the related studies focused on the relationship among AR levels in the primary tumor, clinical characteristics, and disease outcome. AR is widely expressed in EC and its expression in metastatic is significantly higher than that in primary tumors [41]. Most of the studies accessing the prognostic value of AR have found that AR expression may be a favorable prognostic indicator. AR expression correlated with lower histological grade, absence of lymphovascular invasion, a lower proliferation index (Ki67), increased disease-free survival, and overall survival [26, 41–43]. There also exist some studies drawing opposing conclusions [37, 39]. The difference among studies may be explained by the following factors, including ⑴ differences in sample size, length of follow-up and prior treatment regimens; ⑵ use of different antibodies and detection techniques; ⑶ use of different cut-off scores to define AR positivity. Existing shortcomings in these studies limit their use in accessing the prognostic value of AR.

In mouse models of type I EC, short-term enzalutamide treatment, an inhibitor of AR signaling, reduced endometrial tumor burden and increased cancer cell apoptosis in a dose-dependent way. However, enzalutamide increased the incidence of invasive and metastatic tumor [44]. This study suggested that AR signaling may have both oncogenic and tumor-suppressive roles. Oncogenic role of AR may be more involved in EC initiation. Later stages of invasion and metastasis in EC may be partly due to inactivation of cancer suppressive AR signaling.

## What makes the relationship between androgens and EC difficult to assess?

In the circulation, testosterone is highly protein-bound, with a small fraction dissolved freely. The rest of testosterone is bound to sex hormone-binding globulin (SHBG) and albumin. The SHBG-bound fraction is biologically inactive because of the high binding affinity of SHBG for testosterone [45]. Total or free testosterone seems not to be perfectly indicative of the bioactive testosterone concentrations. Some studies have suggested an inverse association of SHBG levels with EC [4, 46]. If subtracting SHBG-bound fraction from total testosterone level, the association between remaining androgens and EC risk is not clear. It has been observed a protective effect of bioavailable testosterone (the component of testosterone calculated to be unbound to SHBG) in estrogen receptor-negative breast cancer among postmenopausal women [47].

Another obvious influential issue is that hyperandrogenism is often combined with several components such as insulin resistance (IR) and visceral adiposity [48–50], which contribute to the development of EC. The metabolic syndrome is characterized by a constellation of symptoms and clinical features, including glucose intolerance, IR, central obesity, dyslipidemia, and hypertension [51]. The association between metabolic syndrome and EC has been observed (reviewed by Yang) [52]. It is difficult to assess which is more important in increasing the likelihood of endometrial carcinogenesis, whether androgen itself or androgen-associated comorbidities. For example, it is noted that testosterone was associated with increased EC risk for women with BMI over 24 kg/m^2^ [48]. After adjustment for BMI and other confounding factors, a decrease in the effect of testosterone was observed [4].

Androgens exert their effects on many cellular targets, including cells of the inflammatory and immune system. A comprehensive review showed that inflammatory cytokines were associated with an increased risk of EC. And they could activate the PI3K/Akt/mTOR signaling pathway that is the most frequently altered pathway in EC [53]. It has been demonstrated that androgens could blunt CD4 + T-cell (Th1)-driven inflammatory response in the vagina while promoting Th2/M2 [54]. Th2/M2 could also be observed at precancerous stage of cervical cancer development [55].

## Concluding remarks

Unfortunately, we are unable to conclude that androgens are the killer or helper in EC. This condition is complex. There are different kinds of androgenic hormones, and different kinds of androgens may have different effects. Elevated androgens (especially testosterone) in EC patients mean that they maybe the potential inciting factor of EC. However, androgen-specific biases in metabolism and the expression of AR may contribute to the different prognosis of patients with EC. For example, 5α-Reds and 17βHSD2 pathway may inhibit the progression of EC mediated by DHT, but aromatases stimulate further progression of EC. However, the downstream signaling pathways have remained unknown. Further experimental studies are clearly required to uncover the impact of related enzymes and AR on carcinogenesis in endometrium.
